# Acute Oral Toxicological Profile of *Croton membranaceus* Mull. Arg. Aqueous Stem Extract, a Herbal Treatment for Benign Prostate Hyperplasia, in Male Sprague–Dawley Rats

**DOI:** 10.1155/2024/7526701

**Published:** 2024-03-07

**Authors:** Daniel Kwame Afriyie, Elvis Ofori Ameyaw, Isaac Tabiri Henneh, George Asare, Ebenezer Ofori-Atta, Seth Kwabena Amponsah, Regina Appiah-Opong

**Affiliations:** ^1^Department of Biomedical Sciences, School of Allied Health Sciences, University of Cape Coast, Cape Coast, Ghana; ^2^Department of Pharmacotherapeutics and Pharmacy Practice, School of Pharmacy and Pharmaceutical Sciences, University of Cape Coast, Cape Coast, Ghana; ^3^Department of Medical Laboratory Sciences, University of Ghana, Accra, Ghana; ^4^Department of Clinical Pathology, Noguchi Memorial Institute for Medical Research, College of Health Sciences, University of Ghana, Accra, Ghana; ^5^Department of Medical Pharmacology, University of Ghana Medical School, Accra, Ghana

## Abstract

*Croton membranaceus* Mull. Arg. is a traditional medicinal plant frequently employed in Ghana for the treatment of benign prostatic hyperplasia and prostate cancer. The objective of this study was to determine the acute oral toxicity of the aqueous stem extract of *Croton membranaceus* (CMASE) in male Sprague–Dawley (S-D) rats. The acute toxicity of CMASE was evaluated using S-D rats randomly divided into four groups of five animals each. Three groups (low dose, median dose, and high dose) of rats received single oral doses of CMASE (1000, 2500, and 5000 mg/kg body weight, respectively) using an oral gavage. The control group was given distilled water. After 14 days of daily observations, hematological, biochemical, and histopathological analyses were conducted on the rats. From the results obtained, doses of CMASE up to 5000 mg/kg did not cause death or induce any clinical indications of toxicity during the study period. Also, the mean body weight and the hematological indices assessed were not significantly affected by the various doses of CMASE compared to the control group. However, serum uric acid and creatinine levels decreased significantly (*p* < 0.001) 14 days after the extract administration. Serum liver function enzyme levels, including alkaline phosphatase (ALP), alanine aminotransferases (ALT), and aspartate aminotransferases (AST), and serum proteins (total proteins and albumin) exhibited significant (*p* < 0.001) non dose-dependent changes (increases and decreases) in treated groups compared to the controls. Other biochemical indices, however, did not differ significantly between the treated groups and the controls. The gross pathological and histological analysis of the heart, liver, and kidney tissues did not reveal any significant changes in histoarchitecture. The oral LD_50_ of CMASE in rats was greater than 5000 mg/kg, indicating that the extract was relatively safe. It must, however, be used with care as a substitute for the roots.

## 1. Introduction

The global incidence of benign prostatic hyperplasia (BPH) has increased significantly from 51.1 million cases in 2000 to 94 million cases in 2019 [1]. Typically, the onset of BPH symptoms occurs in middle-aged men (35 years or older), and by age 50, an estimated 50% of men have been diagnosed with the condition; thereafter, the incidence of BPH cases increases linearly with age [2]. BPH prevalence in Africa and West Africa is believed to be very high despite the fact that there is paucity of data to support this claim. Among the few reports from the continent is one from South Africa which indicates a prevalence of over 50% in adult males of over 60 years old [3]. Also, a study in a rural setting in Nigeria found 23.7% prevalence among male adults [4]. In a Ghanaian study, the prevalence of digital rectal examination- (DRE-) detected enlarged prostates in 950 men aged 50–74 was found to be 62.3% [5]. All these data suggest a high prevalence of BPH on the African continent.

BPH is known to be caused by an imbalance in the proliferation of epithelial and stromal cells in the prostate, which occurs in aging men [6, 7]. Tamsulosin and alfuzosin (alpha-1 blockers), in conjunction with traditional 5*α*-reductase inhibitors (finasteride and dutasteride), have been the mainstay of pharmacotherapy for BPH over the years [8]. However, the use of these conventional drugs has been limited by their numerous side effects, which include dizziness, postural hypotension, headache, tachycardia, abnormal ejaculation, and somnolence, among others [9]. This has resulted in increased patronage of medicinal plant alternatives, which are considered to be generally safer, easily assessable, and affordable [10]. One of these plant alternatives is *Croton membranaceus* Mull. Arg. (family, *Euphorbiaceae*).

*Croton membranaceus* is a well-known traditional medicinal plant in Ghana and the West African subregion for its antibenign prostatic hyperplasia and antiprostate cancer properties from its root extracts [11, 12]. The plant belongs to the genus Croton and is one of the 1300 species in the family *Euphorbiaceae* [13]. Earlier studies on the root bark of the plant revealed that it contained scopoletin and julocrotine [14], with the molecular structure of the former constituent resembling terazosin, a synthetic alpha-1 blocker used conventionally for the relaxation of smooth muscles around the urethra in BPH management [15].

Owing to the growing interest in the medicinal properties of *C. membranaceus,* tremendous scientific studies have been undertaken over the past decades on its root extracts, following the initial studies by Aboagye [12] that sought to establish whether it possessed 5-alpha reductase inhibitory activity. The aqueous root extracts of *C. membranaceus* have been subjected to acute and subchronic toxicity tests, which have validated their safety as well as their anti-ischaemic and anti-antherogenic potentials [16, 17]. There have also been some toxicity studies on the leaves and stem extracts of the plant [17–19]. But it is important to note that, aside from the study by Asare et al. [17]; who conducted a comprehensive toxicological assessment on the root of the plant that encompassed hematological, serum biochemical, and histopathological evaluations, all other previous acute toxicity studies on the stem or root extracts of *C. membranaceus* concentrated on the observation of only clinical symptoms of toxicity. It is worth noting that not all hematological, serum biochemical, and histopathological abnormalities are clinically manifested [20]. This implies that although a test agent may not exhibit clinical signs of toxicity, there could be serious abnormalities in internal vital organs, thus leading to death in the long term. It is important to suggest that test agents that have been declared safe based on only clinical signs of toxicity should be subjected to a detailed toxicological assessment for optimum safety of consumers.

Due to increased demand for the root extract for BPH management in Ghana and the West Africa subregion, there have been undocumented reports of the replacement of root parts with the stem or a mixture of the stem and roots for improved yield without recourse to toxicity evaluation of the stem extract. It is worth noting that the medicinal and/or toxicological effects of one part of a plant cannot be extrapolated to the other part of the plant since different plant parts are known to contain different secondary metabolites in different concentrations [21, 22]. It is very vital for the toxicity profile of medicinal plant extracts to be established before they are marketed as “safe” natural products [23]. The study, therefore, aimed at providing an in-depth acute toxicological evaluation of the aqueous stem extract of *C. membranaceus* for future preclinical studies.

## 2. Materials and Methods

### 2.1. Plant Collection and Extraction

The collection of the plant and extraction procedure has been described previously [24]. Whole plants of *C. membranaceus* were collected during the morning in the month of December, at Mampong-Akwapem (5°35′28″N, 0°14′59″W) in the Eastern region of Ghana. Taxonomists at the Center for Plant Medicine Research (CPMR) verified its authenticity. A voucher specimen of the plant (UCCG-DCOP-007) was deposited at the University of Ghana's herbarium.

The extract was prepared using the modified validated procedure by Afriyie et al. [16]. The stem of the plant were carefully cleaned and allowed to air dry for three weeks. The dried stems were then pulverized into a powder using a milling machine, and the powdered material was sealed in zip-lock bags with labels and kept at room temperature (25°C). Then, 1 kg of the powdered plant part was cold macerated for 24 hours with 4 L of distilled water and heated on a water bath at 100°C for 1 hour. After cooling, the extract was filtered through sterile gauze. The second and third extracts were prepared by repeating this procedure with 3 L of distilled water and then cooling them. After gathering the extracts, they were freeze-dried. Before being used for the in vivo experiment, the percentage yield of the freeze-dried material was determined to be 1.78% using the following formula:

(1) $$\begin{array}{c} \text{Percentage yield}=\frac{\text{Weight of dried extract}\left(g\right)}{\text{Weight of dried powdered plant sample}\left(g\right)}\times 100. \end{array}$$

The freeze-dried extract was then kept in air-tight amber-colored containers and immediately kept in a refrigerator between 2 and 8°C. The aqueous stem extract of *C. membranaceus* (CMASE) procedure was selected based on folkloric preparation protocols.

### 2.2. Experimental Animals

For this study, healthy male Sprague–Dawley (S-D) rats (6-7 weeks old) weighing 110–130 g were used. Rats were purchased from the Animal Experimentation Unit of the Noguchi Memorial Institute for Medical Research. The animals were acclimatized for five days before the experiment, and they were kept at room temperature of 25 ±1°C and at a humidity of 50–60%. They were also maintained at 12 hour light/day cycle. They were fed with conventional laboratory rat diet (Agricare, Kumasi, Ghana) and were given free access to clean water. The animals were given a 12 hour fast before treatment after which the laboratory conditions continued throughout the experiment. The study strictly followed the guidelines outlined by the National Institute of Health's “Guide for the Care and Use of Laboratory Animals.” Ethical clearance was obtained from the Institutional Animal Care and Use Committee at the Noguchi Memorial Institute for Medical Research's Department of Animal Experimentation, University of Ghana with approval number (UG-IACUC 003/18-19).

### 2.3. Acute Toxicity Study

The experimental procedure used in conducting the experiment has been described previously [24]. The acute toxicity study was performed following the Organisation for Economic Co-operation and Development Guidelines for Testing Chemicals with little modifications (OECD 420, 2001). A total of 20 healthy S-D male rats were randomly divided into four groups (*n* = 5/group). Briefly, instead of the recommended doses of 50, 100, 300, and 2000 mg/kg, higher doses of 1000 to 5000 mg/kg were used. This was due to the fact that earlier studies conducted on the root used doses as high as 3000 mg/kg and no death was recorded at such a high dose [17]. The distinct, permanent color markings on the head, tail, back, right foreleg, and left hind leg allowed researchers to identify the rats in each group. The three groups of rats (low dose (LD), median dose (MD), and high dose (HD)) each received a single oral dose of 1000, 2500, and 5000 mg/kg extract, respectively. Distilled water was administered orally to the control group. The rats were observed for clinical toxidrome signs in the form of altered skin, fur, eyes, respiratory pattern, movement, and mucous membranes at 0, 1, 3, and 6 hours postextract administration, as well as every day up until the 14th day. Daily food and water consumption of rats was monitored. Furthermore, body weights of the rodents were documented on days zero, seven, and fourteen subsequent to the extract's administration. On the fifteenth day, the rats were anesthetized with pentobarbitone 50 mg/kg i.p. Blood was collected via cardiac puncture into EDTA (ethylenediamine-tetraacetic acid)-2K tubes for hematological analysis, and portions were collected into gel-separator tubes for biochemical analyses. Afterwards, the rats were euthanized and vital organs/tissues such as the liver, heart, kidney, and prostate were harvested for histopathological analysis. Carcasses of the animals were buried in a designated area following local institutional ethical guidelines as well as the “Guidelines for the Euthanasia of Animals” published by the American Veterinary Medical Association [25].

#### 2.3.1. Hematological Analysis

Hematological parameters including total red blood cells (RBC), total white blood cells (WBC), hemoglobin levels (HGB), packed cell volume (PCV), hematocrit (HCT), platelet counts (PCT), mean cell hemoglobin concentration (MCHC), mean cell volume (MCV), and mean corpuscular hemoglobin (MCHC) were analyzed according to Baker et al. [26].

#### 2.3.2. Biochemical Assessment

After being collected into gel separator tubes and letting them clot for 30 minutes,blood samples were processed for serum by centrifuging (using HUMAX-K, Human-Germany) them for 5 minutes at 3000 rpm. Before use, Sera were kept at −20°C. Using the Selectra Junior Autoanalyzer (Vital Scientific Bv, Version 04, Netherlands), serum samples were examined for a variety of biochemical markers, including total protein, albumin, total bilirubin, alkaline phosphatase (ALP), alanine aminotransferase (ALT), aspartate aminotransferase (AST), total cholesterol, urea, lactate dehydrogenase (LDH), creatine kinase-R (CK-R), and lipids.

#### 2.3.3. Histopathology

The liver, heart, kidney, and prostate tissues were harvested for histopathological examinations. The weights of the harvested organs were determined using a standard weighing balance after cleaning and washing with normal saline and drying with blotting paper. The organs were subsequently processed for histopathological studies after being fixed with 10% buffered formalin. Hematoxylin and eosin (H&E) was used to stain the sectioned tissues (5 *µ*m) of these organs that were embedded in paraffin. Slides were examined using an Olympus CX23 light microscope (model CX23LEDRFSI, China).

### 2.4. Statistical Analysis

GraphPad® Prism Software Version 9.3.1 (San Diego, California, USA) for Windows® 10 was utilized in the analysis of data and plotting of graphs in this study. Results were presented as mean ± the standard error of the mean (S.E.M.). To establish a significant difference analysed parameters between the treatment groups and control group, either a one-way ANOVA followed by Dunnet's *post hoc* test or two-way ANOVA followed by Tukey's *post hoc* multiple comparison analysis was used. *P* values less than 0.05 considered statistically significant.

## 3. Results

### 3.1. Animal Survival and Clinical Observations

**Figure 1:**
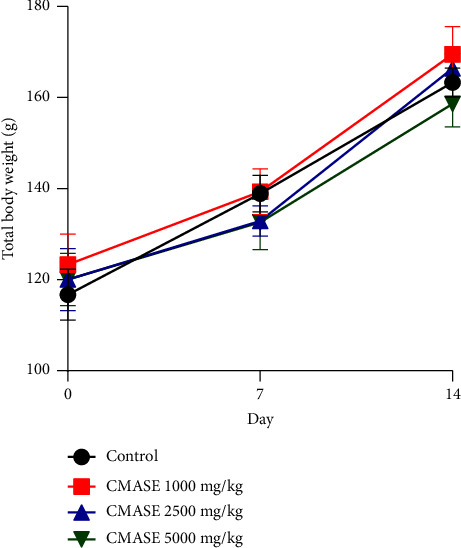
Effect of extract of CMASE on the body weights of male S-D rats 14 days postoral administration of test agents. Data are presented as mean ± SEM. *n* = 5. There were no significant differences between treatment and control using two-way ANOVA.

Results obtained from the study, as presented in Figure 1, indicate that treatment of the rats with various doses of CMASE did not cause death in any of the treated animals. Also, none of the treated groups experienced any toxicity-related symptoms such as restlessness, grooming, tremors, convulsions, pupillary dilation, pinna reflex, salivation, or lacrimation. In general, all groups experienced an increase in body weight at the end of the 14-day experiment. However, rats given the aqueous stem extract of *C. membranaceus* (1000, 2500, and 5000 mg/kg) did not gain any body weight that was significantly different from control rats (*p*  >  0.05). The low and median dose groups experienced the greatest weight gains, whereas the high dose group experienced the least weight gain.

### 3.2. Hematological Analysis

Table 1 presents comprehensive findings on the effects of acute CMASE administration on hematological indices using S-D rats after 14 days. Results generally showed no significant differences in hematological indices between the CMASE treated and control groups (*p*  >  0.05). Comparing the treated rat groups to the control, there was typically no dose-dependent pattern of the effect of different doses of CMASE on the hematological parameters. HGB, RBC, WBC, HCT, MCV, and MCH values were generally slightly lower in the low and median groups than in the control group, whereas the high dose groups' values were slightly higher than their respective controls. The mean MCHC, LYM%, and LYM count (#) values were marginally higher in the treated groups than the values obtained in the control group.

### 3.3. Biochemical Analysis

Table 2 provides information on CMASE's effects on S-D male rats' biochemical parameters at the conclusion of the study. Regarding these indices: gamma-GT, total bilirubin, direct bilirubin, indirect bilirubin (I-BIL), total cholesterol (TC), total triglycerides, HDL, LDL, and urea values, no statistically significant (*p* > 0.05) differences between serum levels among the treated groups and controls were found. Additionally, neither a general nor a dose-dependent pattern of CMASE's effects was seen in these treated groups.

All of the examined liver enzyme indices (ALT, AST, and ALP) showed significant (*p* < 0.001) differences between the treated and control groups. No dose-dependent patterns of CMASE's impact on these liver indices were observed. Also, significant (*p* < 0.001) differences were found between the mean serum values obtained for total proteins, globulins, and albumin between the treated and control groups. Although some treated groups showed significantly lower levels of TP and ALB compared to controls, the values obtained for these indices in the treated groups did not exhibit any dose-dependent pattern. In general, the mean lipid indices for TG, TC, HDL, and LDL obtained in the low and median dose groups were lower than their corresponding control mean values, whereas the high dose values for these indices were marginally higher than their corresponding control group mean values. The mean serum levels of uric acid and creatinine were significantly lower in the treated groups when compared to the control group with regard to the kidney function indices (*p* < 0.001).

### 3.4. Effect of CMASE on Creatine Kinase and Lactate Dehydrogenase

**Figure 2:**
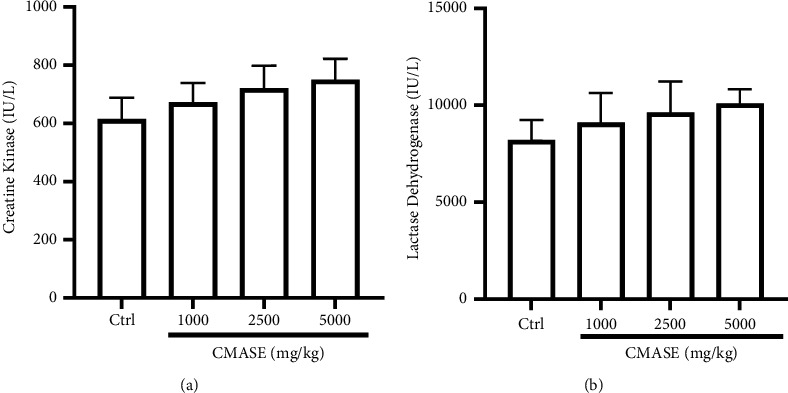
Effect of single-dose administration of CMASE (1000, 2500, and 5000 mg/kg) on serum creatine kinase and lactate dehydrogenase values in S-D rats on the 14th day. Data are presented as mean ± SEM (*n* = 5). There were no significant differences between treatment and control using one-way ANOVA (*p* > 0.05).

Fourteen days following the single-dose administration of CMASE, serum levels of lactate dehydrogenase and creatine kinase in the treatment groups were not significantly (*p* > 0.05) altered compared to the control group. However, a slight elevation of serum levels for these indices was seen with increasing doses of CMASE.  Figure 2 displays the mean serum values for lactate dehydrogenase and creatine kinase.

### 3.5. Histopathology and Relative Organ Weights

#### 3.5.1. Organ Weights of Male S-D Rats

The mean organ weights of the liver, heart, and kidney of the treated groups compared to the control group were not significantly different when they were examined 14 days postacute dose administration of CMASE. Additionally, CMASE had no dose-dependent effects on the organ weights of any of the treated groups (Table 3).

#### 3.5.2. Gross Pathological and Macroscopical Examination

Macroscopically, the kidneys of the CMASE-treated rats were not significantly different from the control group rats. They all had a reddish brown and bean-shaped appearance. Also, the liver of both control and treated group rats appeared normal with smooth, firm consistency and reddish brown in color. Similarly, macroscopical features of the heart had a conical-shaped reddish-brown appearance in control and treatment group rats.

**Figure 3:**
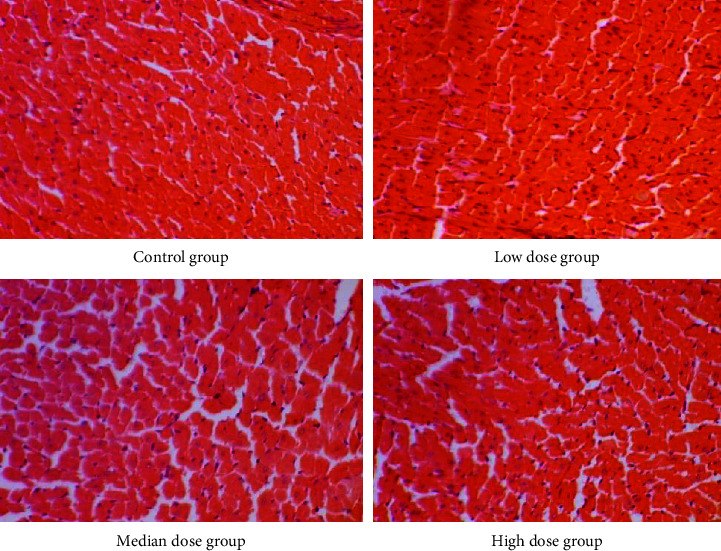
Photomicrograph of cross-section of the cardiac muscle in S-D male rats of the control, low, median, and high dose groups showing normal features of myocardium (H&E stain, ×40).

**Figure 4:**
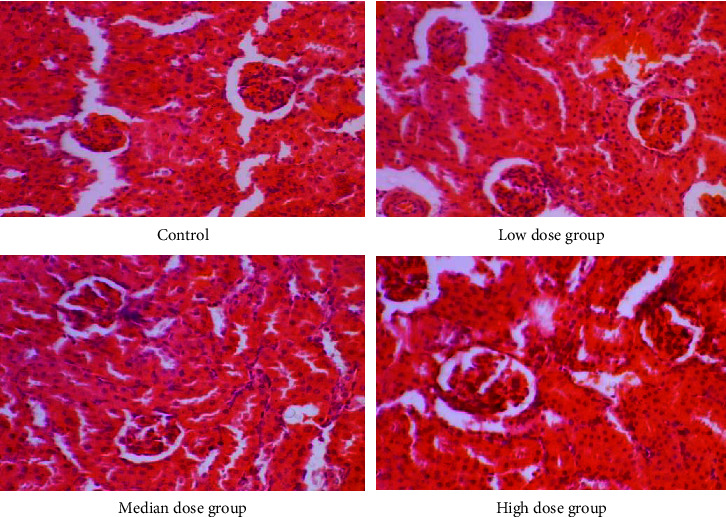
Photomicrograph of S-D rat kidney showing the normal architecture of the glomeruli and tubules of the control low, median, and high dose group animals (H&E stain, ×40).

**Figure 5:**
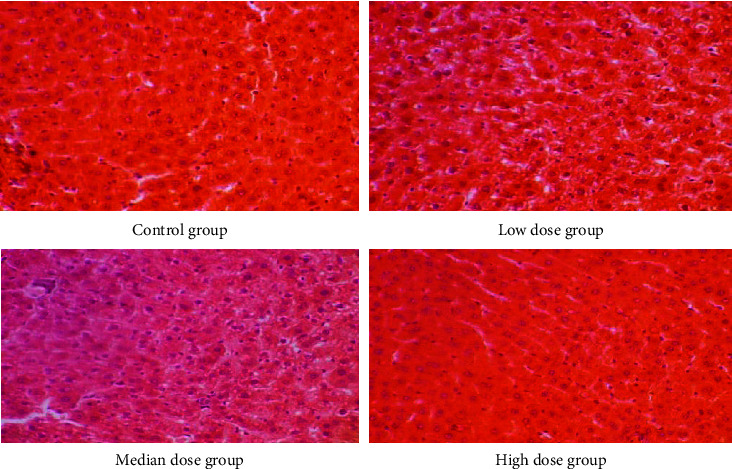
Photomicrograph of cross-section of the hepatic tissue in S-D rats showing normal features of hepatocytes in the control, low, median, and high dose groups animals (H&E stain, ×40).

Gross anatomical features of the heart, liver, and kidneys of rats in the CMASE-treated groups were not markedly different from those in the control group. No serious abnormalities or damages were observed in any of the organs examined. Sections of these organs were further examined histologically, most organs showed normal morphological and staining features, with few mild pathological lesions. Where pathological lesions were present, they were mild and potentially reversible. Heart tissue sections from both the treated and control groups were normal without any infarcts or leucocyte infiltrations (Figure 3). Also, sections from the kidneys of the treated and control groups displayed normal renal tissue, including dilated tubules, a few congested renal tissues, and no tubular or glomerular necrosis (Figure 4). Moreover, sections from the liver of both treated and control groups showed only a few spots of dilated central veins, but there were no hepatocellular necrosis in the tissues (Figure 5). In summary, the liver, kidney, and heart of both the treated and control groups showed similar morphological features histologically, with very little or no evidence of CMASE-induced damage.

## 4. Discussion

Ethnomedicinal plants have been used in folk medicine to treat various ailments. Their natural origin has fueled the erroneous assumption that they are very safe and devoid of possible serious adverse effects [10, 27]. To guarantee their safety for human consumption and marketing, medicinal plant extracts and other potential drug molecules must undergo toxicological and pharmacological evaluations as required by regulatory agencies. The initial test conducted to gather information on the hazardous properties of a substance is the acute oral toxicity test. This test enables the substance to be categorized and classified based on the Globally Harmonized System (GHS) for chemicals causing acute toxicity [28]. One of such medicinal plants that have been used extensively for the treatment of benign prostate hyperplasia is the *Croton membranaceus* despite its limited toxicological assessment, especially for the stem.

Oral administration of single high doses of *Croton membranaceus* aqueous stem extract, CMASE (1000, 2500, and 5000 mg/kg) in this study to male S-D rats was found to be almost nontoxic to the animals. In fact, no signs of behavioral or clinical toxicity or mortality were seen during the study's time frame. The present study therefore suggests that the aqueous stem extract falls under the Globally Harmonized Classification System (GHS) unclassified category and that the LD_50_ of the extract is greater than 5000 mg/kg [28]. Results from this section of the study were comparable to those from related studies on acute toxicity that used *C. membranaceus* root extracts [17–19]. In the aforementioned studies, no serious signs of toxicity or death were recorded for the extracts at doses as high as 3000 mg/kg. Thus, the stem, root, and leaves of *Croton membranaceus* could be said to nontoxic even at high doses.

Upon exposure to toxic substances, abnormalities in body weight and internal organs are among the most important sensitive indicators of toxicity that are mostly assessed [29, 30]. Studies have revealed that abnormal reductions in body weights of experimental animals could be suggestive of probable side effects or adverse reactions to test substances [31, 32], and reductions of initial weights beyond 10% are indicative of severe toxicity [33]. In this study, there were no appreciable differences in the body weights of rats in the treated and control groups. Importantly, all groups of rats gradually grew in weight, and this could be attributed to the fact that the animals had regular dietary and hydration habits throughout the 14-day study period. The extract's potential for safety was indicated by the observations made regarding how acute doses of CMASE did not significantly affect body weights as well as food and water intake compared to the control group. The extract is therefore not likely to exert any deleterious effects on the metabolic growth or health of consumers.

The results also suggest that there were no significant differences in the mean organ weights of the liver, heart, and kidney between the treated groups and the control group when examined 14 days after the acute dose administration of CMASE. Significant changes in vital organ weights of treatment groups compared to the control group gives an indication of toxicity of test agents [34]. In this study, the absence of significant alterations in organ weights suggests that CMASE administration at the tested doses did not induce any overt adverse effects on these organs. However, according to OECD [28], further investigations involving hematological, biochemical, and histopathological assessments need to be conducted to ascertain the toxicity profile of the extract.

Analysis of hematological indices such as HGB, WBC, RBC, and HCT aids scientists and clinicians in diagnosing the presence of anemia, while other related indices such as reticulocyte count, MCV, MCH, and MCHC are used to distinguish the type of anemia [24, 35]. Toxicological risk of blood parameters is vital because the bone marrow, being part of the hematopoietic system, is a target tissue for toxic compounds [29]. Consequently, it offers sensitive predictive values for toxicity in humans using assays that involve rodents and other animals [36]; Adeneye et al., 2006; Olson et al., 2000. In this study, the hematological parameters (RBC, WBC, PLT, HGB, HCT, MCV, MCH, and MCHC) were compared between the control and CMASE-treated groups, and it was found that CMASE was not toxic to the hemopoietic system because there were no significant differences in these indices between the control and treated groups. These results agree with an earlier study by Asare et al. [17], who studied the aqueous root extract of the plant. It was observed that the administration of the extract does not adversely affect the hematopoietic system of rats. Another closely related study on the stem extract of the plant only reported on clinical signs of toxicity without recourse to alterations in biochemical parameters [19], and that underscored the need for the present study. Another important observation was that most of the hematological parameters were lower in the median dose compared to the low dose and the high dose groups, though insignificant. This could be explained by the fact that hematological responses can vary among individual animals, even when exposed to the same dose. This variability may lead to unexpected results where the median dose, which represents the middle value in a set of doses, happens to elicit lower responses compared to the lowest dose and the highest dose in some individuals [37].

According to Gowda et al. [38], significant increases in blood levels of creatinine, uric acid, and urea are signs of either abnormal kidney function or other diseases like glomerulonephritis, shock, congestive heart failure, polycystic kidney disease, acute tubular necrosis, and dehydration. However, there were significant differences in the levels of these biochemical parameters in the CMASE-treated groups compared to the control group in terms of creatinine and uric acid levels. Decreased serum creatinine levels mostly occur in subjects with muscular dystrophy paralysis, anemia, leukemia, and hyperthyroidism [39]. The comparison with the study by Asare et al. [17] who assessed the root extract of the plant provides valuable context for interpreting the findings of the present study. While the earlier study observed no significant changes in renal function parameters, such as urea and creatinine, with doses of 1500 and 3000 mg/kg of the root extract of the plant, the present study found a significant reduction in creatinine levels at doses ranging from 1000 to 5000 mg/kg of the stem extract. This discrepancy in findings suggests that different parts of the plant or different extraction methods may yield varying effects on renal function parameters. In addition, it underscores the importance of considering the specific dosage and formulation used when assessing the safety profile of medicinal products derived from natural sources. The significant reduction in creatinine levels observed in the present study at higher doses of the stem extract raises concerns regarding its potential impact on kidney function, particularly in individuals with pre-existing kidney impairment. Therefore, caution is warranted when using medicinal products containing these extracts, especially for individuals with renal dysfunction.

The liver is a major target and organ for drug bio-metabolism, so evaluation of liver indices is used as an indicator of the potential toxicity of test substances [40, 41]. Studies have demonstrated that hepatotoxic drugs or extracts may cause liver damage that results in increased ALT, AST, and total protein levels [29, 42]. ALT is found in the liver and is therefore a much more sensitive marker for liver toxicity, as opposed to AST, which can also be found in the myocardium, skeletal muscles, brain, and kidney. The study's findings demonstrated that acute oral administration of CMASE at doses between 1000 and 5000 mg/kg caused a significant alteration in the serum levels of ALT, AST, and total proteins in the treated groups. This may suggest that the liver is a potential target organ for toxicity. However, on account of the fact that the histology did not show any abnormalities, hepatotoxicity is unlikely It is important to state that the results obtained differed from that obtained by Asare et al. [17], who found that the root extract of the plant did not cause significant alterations in AST or ALT levels in the serum of rats used.

The observation that median doses resulted in higher levels of AST, ALT, and ALP compared to both the high and low doses, despite all doses being significantly higher than the normal control, suggests a complex relationship between dose and the biochemical response. One possible explanation for this phenomenon is the biphasic or nonlinear nature of the dose-response curve for these parameters. In other words, the biochemical response may not increase linearly with increasing doses but rather reach a peak or plateau before declining or stabilizing at higher doses. This could be due to various factors, including saturation of metabolic pathways, receptor binding, or toxicological effects [37]. In addition, it is important to consider the mechanisms underlying the regulation of these enzymes in the body. AST, ALT, and ALP are produced primarily in the liver and are released into the bloodstream when liver cells are damaged or stressed. At lower doses, the body may be able to compensate for the increased enzyme production and maintain homeostasis to some extent. However, at higher doses, the cellular damage or stress may exceed the body's compensatory mechanisms, leading to a disproportionate increase in enzyme levels, thus accounting for the disparities between responses from the median and highest doses. Aronson and Ferner [37] also argued that in such circumstances, the observed effect may not be real, as it could have arisen due to random chance or methodological issues like bias or confounding factors. Additionally, individuals vary greatly in their susceptibility to experiencing adverse reactions to test agents. Further investigations are therefore warranted to confirm the observation which also occurred in other biochemical parameters such as urea, uric acid, and albumin.

Moreover, the liver is a major site of protein synthesis, and reductions in serum levels of total protein, albumin, and globulin could be suggestive of impaired hepatocellular function or hepatocyte damage [43]. The study revealed a significant difference (*p*  <  0.001) in serum levels of ALB and TP only between the low dose and control groups. The results obtained deviated from an earlier which reported that total proteins, albumin, and globulin were not affected by low or high dose of root extract of *Croton membranaceus* administration [17]. Interestingly, it could be observed from the study that histological examination of the liver did not reveal any alteration of the histoarchitecture or damage.

Triglycerides, total cholesterol, LDL, and VLDL are the most common lipids, and levels that are higher than the normal range suggest a higher risk of atherosclerosis, heart disease, stroke, and other cardiovascular diseases [44]. Although the 1000 and 2500 mg/kg doses of CMASE appeared to have marginally reduced lipid parameters, the extract generally did not alter these parameters significantly, which indicates that its acute oral doses do not interfere with lipid metabolism adversely or induce cardiovascular diseases.

Elevated serum enzyme levels of LDH and CK are biomarkers of heart or muscle damage [45]. Therefore, the levels of CK or CK-MB and LDH in toxicity studies have been used clinically to evaluate the degree of damage to the heart, skeletal muscles, and brain [46–48]. The results of the current study suggest that acute oral doses of CMASE (C. membranaceus aqueous stem extract) did not cause any observable changes in the levels of creatine kinase (CK) and lactate dehydrogenase (LDH) in the serum. These markers are commonly used to assess potential toxicity to the brain, heart, or skeletal muscles. The lack of significant changes in CK and LDH levels implies that CMASE may not exert toxic effects on these organs and tissues when administered orally. Comparing these findings with a previous study conducted by Asare et al. [17], which investigated the effects of the root extract of the same plant, some differences were noted. Specifically, Asare et al. found that LDH levels were unaffected by the root extract, similar to the current study. However, they observed that CK and CK-MB levels were significantly lower in the groups treated with the root extract compared to the control group. The juxtaposition of these studies suggests that different parts of the plant (stem extract vs. root extract) may have varying effects on cardiac markers such as CK and CK-MB. While the stem extract in the current study did not significantly alter CK and LDH levels, the root extract in the previous study led to lower CK and CK-MB levels. This highlights the importance of considering the specific plant part and its extraction method when assessing potential physiological effects. In this study, no gross lesions were found in the tissues of the heart, liver, or kidney compared to the control group organs in the gross pathological and histopathology examinations. No significant pathological lesions were found in the kidneys or livers of the treated groups despite some significant changes in some renal and liver function serum parameters. This could be due to the fact that many nephrotoxic plants, animal poisons, medications, chemicals, and illicit drugs can induce some level of acute kidney injury by varying the pathophysiological pathways and/or biochemical parameters without necessarily causing acute structural changes in the kidney [49]. It is, therefore, important to note that the correlation between biochemical parameters and histological findings is complex, and various factors can influence the observed patterns. In clinical practice, a comprehensive assessment of kidney health often involves the integration of both biochemical and histological information, along with clinical context, to provide a more accurate understanding of renal function and potential pathology. The same applies to other organs studied in which biochemical alterations did not manifest in structural changes of the organs.

Results from this study suggest that CMASE could be regarded as relatively safe following acute exposure and that the LD_50_ value may exceed 5000 mg/kg. This study agrees with an earlier study by Appiah [19], who conducted previous acute oral studies in S-D rats using doses of *C. membranaceus* aqueous stem extract up to 5000 mg/kg in rats but reported only on clinical observations. Asare et al. [17], who also reported on the acute toxicity of the aqueous root extract of the plant, reported an LD_50_ that was greater than 3000 mg/kg. This study also agrees with an earlier study by Sarkodie et al. [18] who reported that the LD_50_ of aqueous leaf extract of *C. membranaceus* was greater than 5000 mg/kg. It is worth mentioning that Sarkodie and his colleagues' [18] study only looked out for clinical signs of toxicity and death without recourse to hematological, biochemical or histopathological investigations. The current study, however, reports on a comprehensive acute toxicity profile of stem extract encompassing clinical signs of toxicity, hematological, serum biochemical, and histopathological toxicity profiling. This, to our knowledge, is the first in-depth acute toxicity study on the aqueous stem extract of *C. membranaceus* in rats. It is still recommended that additional studies involving repeated drug administration (subacute, subchronic, or chronic toxicity) should be conducted.

## 5. Conclusion

The results suggest that oral administration of the aqueous stem extract of *C. membranaceus* at doses up to 5000 mg/kg did not result in mortality in the experimental animals, indicating a relatively low acute toxicity profile. Additionally, various parameters including behavioral observations, total body and organ weights, hematological parameters, and organ histology did not show significant differences compared to the control group, further supporting the overall safety profile of the extract.

However, caution is advised due to alterations observed in certain serum biochemical parameters. While the extract did not cause mortality or significant adverse effects on most physiological parameters measured, changes in serum biochemical parameters indicate potential physiological alterations that warrant attention. These alterations may suggest underlying effects on metabolic processes or organ function that could have implications for long-term health.

Therefore, while the extract appears to be relatively nontoxic based on acute toxicity testing and general physiological parameters, the observed changes in serum biochemical parameters indicate the need for careful consideration when using the extract. Patrons and healthcare providers should be aware of these potential effects and monitor for any signs of adverse reactions or physiological changes when using the extract, particularly at higher doses or with prolonged use.

Further research, including subchronic and chronic toxicity studies, as well as clinical trials in human subjects, may provide additional insights into the safety profile and potential health effects of the aqueous stem extract of *C. membranaceus*. Additionally, investigating the underlying mechanisms of action responsible for the observed alterations in serum biochemical parameters could help elucidate the physiological effects of the extract and inform safer usage guidelines.

## Figures and Tables

**Table 1:** Hematological parameters of S-D rats after 14 days of administration of CMASE.

Hematological parameters	Rat groups				
	Control	Low dose 1000 mg/kg	Median dose 2500 mg/kg	High dose 5000 mg/kg	*P* value
WBC × 10^3^ (*μ*l)	13.42 ± 1.60	12.53 ± 2.21	11.95 ± 1.46	14.39 ± 1.57	0.770
RBC × 10^6^ (*μ*l)	8.35 ± 0.15	7.99 ± 0.29	7.86 ± 0.13	8.57 ± 0.40	0.285
HGB (g/dL)	14.73 ± 0.25	14.14 ± 0.53	14.00 ± 0.31	15.28 ± 0.25	0.077
HCT (%)	47.73 ± 0.73	45.44 ± 2.07	44.50 ± 0.72	48.58 ± 1.68	0.190
MCV (fL)	57.18 ± 1.33	56.92 ± 1.69	56.64 ± 0.8	56.78 ± 1.05	0.992
MCH (pg)	17.63 ± 0.35	17.72 ± 0.49	17.58 ± 0.32	17.88 ± 0.42	0.954
MCHC (g/dl)	30.85 ± 0.22	31.18 ± 0.26	32.48 ± 1.2	31.45 ± 0.15	0.323
PLT × 10^3^ (*μ*l)	767 ± 96.7	576.8 ± 26.85	533.8 ± 50.97	631 ± 29.37	0.059
LYM (%)	68.65 ± 2.23	74.52 ± 3.25	68.06 ± 2.26	71.33 ± 2.39	0.302
LYM (# ×10^3^/*μ*l)	9.25 ± 1.24	9.31 ± 1.75	8.40 ± 0.93	10.38 ± 1.46	0.792

Data are presented as mean ± SEM. *n* = 5. *P* values were obtained from one-way ANOVA (treatment groups compared to control for each parameter). Since, *p* values were greater than 0.05, thus there were no significant differences between treatment and control groups. WBC = white blood cells; RBC = red blood cells; HGB = hemoglobin levels; HCT = hematocrit; MCV = mean cell volume; MCH = mean corpuscular hemoglobin; MCHC = mean cell hemoglobin concentration; PLT = platelet counts; LYM = lymphocytes.

**Table 2:** Biochemical indices of S-D rats treated with acute doses of CMASE after 14 days.

Biochemical parameters	Control	Low dose 1000 mg/kg	Median dose 2500 mg/kg	High dose 5000 mg/kg
T-BIL (*μ*mol/l)	0.60 ± 0.35	0.38 ± 0.16	0.44 ± 0.16	0.35 ± 0.06
D-BIL (*μ*mol/l)	1.28 ± 0.24	1.23 ± 0.16	1.00 ± 0.02	1.51 ± 0.16
IBIL (*μ*mol/l)	0.85 ± 0.25	1.10 ± 0.25	1.14 ± 0.09	0.98 ± 0.19
ALT (U/L)	78.15 ± 0.92	60.75 ± 0.57^*∗∗*^	87.83 ± 0.13^*∗∗*^^**L**^	73.75 ± 0.86^L,M^
AST (U/L)	267.70 ± 0.52	245.30 ± 0.50^*∗∗*^	395.38 ± 0.04^L^	301.90 ± 0.59^L,M^
ALP (U/L)	390.78 ± 0.30	435.55 ± 0.32^*∗∗*^	449.08 ± 0.95^L^	304.75 ± 0.39^L,M^
Gamma-GT	1.38 ± 0.27	1.60 ± 0.52	1.90 ± 0.58	1.75 ± 0.19
TP (g/l)	66.73 ± 0.71	58.55 ± 0.25^*∗∗*^	63.23 ± 0.64^*∗∗*^^**L**^	66.68 ± 0.20^L,M^
ALB (g/dL)	35.05 ± 0.78	29.55 ± 0.98^*∗∗*^	35.40 ± 0.57^L^	34.38 ± 0.59^L^
Glo (g/dL)	31.68 ± 0.89	28.13 ± 0.40^*∗*^	28.85 ± 0.93	33.78 ± 0.97^L,M^
AST/ALT	3.45 ± 0.17	4.53 ± 0.46	5.15 ± 0.67	3.40 ± 0.17
TC (mmol/L)	2.45 ± 0.19	2.09 ± 0.12	1.98 ± 0.17	2.51 ± 0.16
TG (mmol/L)	1.46 ± 0.15	1.27 ± 0.12	1.44 ± 0.14	1.80 ± 0.29
LDL (mmol/L)	0.59 ± 0.09	0.46 ± 0.02	0.45 ± 0.03	0.58 ± 0.06
HDL (mmol/L)	0.99 ± 0.04	0.89 ± 0.04	0.91 ± 0.08	1.09 ± 0.04
CREA (mmol/L)	64.30 ± 0.80	52.39 ± 0.95^*∗∗*^	56.55 ± 0.53^*∗∗*^^**L**^	57.87 ± 0.80^*∗∗*^^**L**^
UREA (mmol/L)	6.15 ± 0.55	6.73 ± 0.32	6.65 ± 0.16	5.34 ± 0.43
URIC ACID (mmol/L)	211.60 ± 0.19	148.33 ± 0.65	175.35 ± 0.02^*∗∗*^	156.23 ± 0.74^*∗∗*^^**LM**^

Data are presented as mean ± SEM. *n* = 5. The symbols ^*∗*^ and ^*∗∗*^ represent *p* < 0.05 and *p* < 0.01, respectively, whereas, ^**L**^*p* < 0.01 compared to the low dose group. ^**M**^*p* < 0.01 compared to the median dose group (two-way ANOVA followed by Tukey's *post hoc* test). HDL = high-density lipoprotein; LDL = low-density lipoprotein; VLDL = very low-density lipoprotein; SEM = standard error of mean; T-BIL = total bilirubin; D-BIL = direct bilirubin; I-BIL; TP = total protein; TC = total cholesterol; ALB = albumin; Glo = globulin; TG = triglycerides; CREA = creatinine; gamma GT = gamma-glutaryl transferase.

**Table 3:** Effect of single-dose administration of CMASE (1000, 2500, and 5000 mg/kg) on organ weights of male S-D rats on the 14th day.

Organ	Control	Low dose	Median dose	High dose	*P* values
Kidney (g)	1.20 ± 0.08	1.24 ± 0.06	1.04 ± 0.08	1.16 ± 0.07	0.97
Liver (g)	6.73 ± 0.19	6.87 ± 0.40	6.67 ± 0.21	7.00 ± 0.52	0.91
Heart (g)	0.62 ± 0.02	0.64 ± 0.04	0.56 ± 0.03	0.60 ± 0.05	0.52

Data are presented as mean ± SEM. *n* = 5. There were no significant differences between treatment groups and control group using one-way ANOVA (*p* > 0.05).

## Data Availability

All data generated or analyzed during this study are included in this published article.

## Abbreviations

CMASE: Aqueous stem extract of *Croton membranaceus*
S-D: Sprague–Dawley
ALP: Alkaline phosphatase
AST: Aspartate aminotransferases
BPH: Benign prostate hyperplasia
OECD: Organisation for Economic Co-operation and Development
EDTA: Ethylenediamine-tetraacetic acid
RBC: Red blood cells
WBC: White blood cells
HGB: Hemoglobin levels
PCV: Packed cell volume
HCT: Hematocrit
PCT: Platelet counts
MCHC: Mean cell hemoglobin concentration
MCV: Mean cell volume
MCHC: Mean corpuscular hemoglobin
LD: Low dose
MD: Median dose
HD: High dose
LDH: Lactate dehydrogenase
CK-R: Creatine kinase-R
H&E: Hematoxylin and eosin
ANOVA: One-way analysis of variance
IBIL: Indirect bilirubin
TC: Total cholesterol
LYM: Lymphocyte
GHS: Globally harmonized system
HDL: High-density lipoprotein
LDL: Low-density lipoprotein
VLDL: Very low-density lipoprotein
SEM: Standard error of mean
D-Bil: Direct bilirubin
TP: Total protein
TC: Total cholesterol
ALB: Albumin
Glo: Globulin
TG: Triglycerides
CREA: Creatinine
DRE: Digital rectal examination (DRE).
